# Influences of Social Capital on Health and Well-Being from Qualitative Approach 

**DOI:** 10.5539/gjhs.v5n5p153

**Published:** 2013-06-25

**Authors:** Ayano Yamaguchi

**Affiliations:** 1 College of Foreign Studies, Reitaku University, Kashiwa, Chiba, Japan

**Keywords:** social capital, health, well-being, cross-cultural views, qualitative study

## Abstract

The social capital surrounding health including health and well-being, the way in which they function as multi-dimensional constructs, and the potential stability of relationships among the social capital were examined across universities in Hawaii and Japan. Maintaining or strengthening social factors of collective and individual health and well-being is a core factor of social capital and is instrumental in reducing worry and increasing trust. Qualitative in-depth interviews with 64 male and female college students (32 college students at the University of Hawaii at Manoa; 32 college students at Reitaku University in Japan) were used to collect information on social capital of health and well-being and associated concepts; students’ perceptions were grouped under 11 themes. The data indicates that social capital has an impact on college students’ health and well-being. They also suggest that differences in health status and well-being can be plausibly attributed to processes associated with socio-environmental circumstances and situations.

## 1. Introduction

This study attempts to develop a theoretical perspective on social factors, such as social capital, that could address individual health status, while at least partially dealing with definition and measurement issues. Social capital is controversial issue, and no consensus exists in the health research literature on their definition or measurement; thus, social capital is subjects that might warrant investigation.

The first main purpose of this study is to gain a grounded understanding of the relationships among significant social factors, including social capital and social environmental factors (e.g., socio-economic and socio-demographic status) and their impact on health and well-being. It is apparent that these social factors are central to one's health and well-being; thus, they are potentially powerful explanatory variables of variations in health and well-being among different samples and populations. However, the literature does not clearly define these explanatory variables nor does it define potentially dependent variables. Because previous studies have found no clear distinction between different forms of health and well-being that can delineate fundamental differences in how people live their lives and pursue gratification, it is important to conduct and pursue systematic research on this topic (Lau & Li, 2011; Morgan, Rivera, Moreno, & Haglund, 2012; Summach, 2011).

The second main purpose of this study is to gain a grounded understanding of whether the relationships among social factors are stable in terms of health and well-being when examined across different cultures. Little research on the distinctions among these relationships across cultures exists. Initially, this study will review the literature regarding the social factors including social capital mentioned above. Two research questions will be addressed (Giordano & Lindstrom, 2010; Oksanen, Kouvonen, Vahtera, Virtanen, & Kivimaki, 2010).

Moreover, health science research has not yet clearly identified whether social capital has any impact on health and well-being in Japanese culture and society. At the individual and ecological levels, some findings have been presented for associations among cognitive social capital, participation, and self-rated health in the Japanese population (Suzuki et al., 2010; Iwase et al., 2010). Although much research identified and clarified the certain findings and implications for the importance of the association between social capital and health status in some social contexts, it still remains unclear and vague whether social capital, particularly individual level social capital, is a determinant of health and well-being in Japanese culture and society and whether Western-derived concepts and measures of social capital can be applied to Japanese culture and society.

For these reasons, in the present study, we used a multilevel perspective to explore new findings pertaining to whether social capital at the individual level is a determinant of health and well-being for the college students in Japan thorough the whole of Japanese culture and society. To explore the effects of goals, the main objective of this study were to understand that illustrates the process underlying the relationship of individual social capital and individual and family characteristics to health and well-being for college students in Japan.

The reminder of this study is organized as follows: the review of literature presents the theoretical development of the proposed model. Then the design and results of a proposed model study testing the proposed model are reported. Finally, this study investigates whether the previous findings about the relationship are confirmed and highlights how social capital affects psychological well-being for adolescents. Based on the findings, theoretical and practical contributions of this study are also discussed.

### 1.1 Conceptualizing Social Capital

Social capital has a long history in the social sciences. It is an umbrella concept that explains institutions, relationships, and norms that shape the quality and quantity of interactions in social relationships. Whether social capital is viewed as an individual or collective property determines the framework for understanding its functionality, which in turn determines the constructs involved and suggests at what level action can be taken to strengthen it (Poortinga, 2006). From an individual perspective, social capital is seen to consist of people's social relations (e.g., egocentric networks) and whether they believe others can be trusted; these link to resources individuals can draw on to help them meet their needs (Giordano & Lindstrom, 2010; Oksanen et al., 2010). Overall, individual and collective perspectives on social capital have certain components in common, although the measurement of these varies based on how they are framed. Putnam (2002) is often cited as defining the key constructs of social capital as networks, norms, and trust, which seem to provide an adequate framework for organizing the research conducted to date, regardless of conceptual approach.

### 1.2 Domains and Dimensions of Social Capital

*Bonding* social capital refers to trusting and cooperative relations among members of a network who see themselves as sharing an identity. It exists in strong ties and interpersonal relationships among friends, family members, and neighbors. It also tends to be concentrated within particular groups, and creates local cohesion, although it can also lead to fragmentation (Poortinga, 2006; Putnam, 2002; Szeter & Woolcock, 2004). *Bridging* social capital, by contrast, refers to relations of mutual respect between people who know that they are not socio-demographically alike (differing by age, ethnocultural group, class, etc). It also exists among people with different backgrounds or relatively weaker ties in terms of trusting relations with those from different demographic groups (Poortinga, 2006; Putnam, 2002; Szeter & Woolcock, 2004). *Linking* social capital pertains to individuals’ overall portfolio of social relationships; it is central to the ability to shape welfare, health and well-being, and also refers to the capacity of available community resources (Poortinga, 2006; Putnam, 2002; Szeter & Woolcock, 2004).

### 1.3 Effect of Social Capital on Health and Well-Being

A great deal of research has been conducted on the relationships among different aspects of social capital and different aspects of health. What follows is a summary of correlations that have been established in the literature. Social capital is positively related with life expectancy and inversely related with mortality. Studies at the state and national levels in the U.S., Europe, Russia, and Africa indicate a strong relationship between social capital and life expectancy and mortality. These studies indicate that social trust and association membership are positively associated with life expectancy and negatively associated with both all-cause and cause-specific mortality (Giordano & Lindstrom, 2010; Lau & Li, 2011; Morgan et al., 2012; Summach, 2011; Oksanen et al., 2010).

Social capital is positively related with health status. Putnam (2002) found a strong linear relationship between the public health index and the social capital index, particularly based on social integration and social support. Several studies indicated that higher levels of community trust were positively associated with self-reported health status (Giordano & Lindstrom, 2010; Lau & Li, 2011; Morgan et al., 2012; Summach, 2011; Oksanen et al., 2010).

### 1.4 Cross-Cultural Applicability and Validity of Social Concepts

This study asks if a measure of key social concept, such as social capital can be found that is universally valid across cultures and cultural contexts. The question leads to examining both the cross-cultural validity of concepts and to the cultural appropriateness of measurement tools. Thus, there are two related issues at stake, the universal applicability and validity of concepts, and second, the relevancy of common or diverse measures (Giordano & Lindstrom, 2010; Lau & Li, 2011; Morgan et al., 2012; Summach, 2011; Oksanen et al., 2010). Therefore, the research questions will be:

RQ1: How are the concepts of health and well-being related to social capital?

RQ2: How stable are these relationships when examined across different cultures?

## 2. Method

The research method reports the major qualitative findings from the results derived from 64 qualitative, in-depth individual interviews (32 interviews from the University of Hawaii at Manoa in Honolulu, Hawaii, and 32 interviews from Reitaku University, Kashiwa City, Japan) that were conducted in early 2009. The qualitative study explored and examined individuals’ daily life experiences in terms of social capital such as social capital and social environmental factors that might be related to health and well-being. The purpose of the interviews was to provide richer details about the impact of significant social capital on health and well-being and to seek fresh insights into aspects that influence individuals to move towards health, well-being, and wellness. In order to create individual interview guidelines, Blum's health and well-being model (1981) was utilized in this study.

### 2.1 Interview Method: Individual Interviews

The Individual Interview Guide was created based on Blum's health and well-being model (1981). The interviews were guided by a checklist (Individual Interview Guide) based on Blum's health and well-being model that focused on five main topics: 1) health status in terms of physical, social, psychological, mental, and spiritual health; 2) environment in terms of achievement, economics, and safety; 3) lifestyle; 4) behaviors; and 5) medical and healthcare services. 32 interviews from the University of Hawaii at Manoa and 32 interviews from Reitaku University in Japan were audio-taped with the consent of the participants and were subsequently transcribed. The demographic information collected from the interviewees revealed that the 32 participants from the University of Hawaii at Manoa had a mean age of 22.2 years (41% male, 59% female), while the students from Reitaku University had a mean age of 20.8 years (47% male, 53% female). The year in school identified by the participants at the University of Hawaii at Manoa was: freshman (34%); sophomore (25%); junior (16%); senior (16%); and graduate (9%). The year in school identified by participants from Reitaku University was: freshman (22%); sophomore (10%); junior (34%); and senior (34%).

### 2.2 Qualitative Data Analysis

According to qualitative data analysis, closed-ended interview questions were used to develop and introduce the main themes of cultural differences between the students at the University of Hawaii and at Reitaku University; these were analyzed using univariate statistics. Results presented in the next section discuss the following: 1) health status in terms of physical health, social health, psychological and mental health, and spiritual health; 2) environment in terms of achievement, economics, and safety; 3) lifestyle; 4) behaviors; and 5) medical care and health care services.

Table 1 shows that health researchers pay attention to study population overall health, including health and well-being and the manner in which the social contexts of Hawaii and Japan and social conditions in each location influence health and well-being. This study can summarize the qualitative findings by saying that, in Hawaii, there was greater trust and a greater sense of community; this suggests that the history of the state and the context of the university may be related to this finding. There was also greater belief in and trust of religion, and participants were more likely to consider themselves “spiritual” when compared to Japanese college students. Finally, there was greater issue whether or not the college students in Hawaii have health insurance to help them maintain their better health status and better level of well-being.

This study can also summarize the qualitative finding that, in Japan, there was a lower level of trust in general and a lower sense of community. This, in turn, leads to higher levels of concern or worry. This study can also summarize the qualitative finding that, in Japan, the lower social capital and distrust, sense of community, and/or feelings of hopelessness may be linked to higher levels of worry or concern, and a lowered sense of “spirituality,” which reflects social changes in Japan over the last 20 years, especially in light of the country's economic troubles. Thus, today's college students do not show the traditional social solidarity and trust which have been traditional, simply because they are less likely to trust people in their society or government; this leads to decreasing levels of social solidarity.

**Table 1:** Qualitative individual interview texts

Social Capial	University of Hawaii at Manoa	Reitaku University
Some relationships, networks, or community ties	97%	100%
Members of groups or organizations	97%	100%
Have a community	81%	56%
Can trust the majority of people	88%	44%

## 3. Results

During individual qualitative interviews, two research questions were asked in order to examine how the concepts of health and well-being were related to other social capital such as social capital, and to explore how stable these relationships were when examined across different cultures. The data, which was drawn from individual interviews, showed important results. The interviews also provided significant insights into the social issues that affected the health and well-being of college students at the University of Hawaii at Manoa and Reitaku University in Japan.

### 3.1 Perception of Trust in Social Capital

#### 3.1.1 Support and Trust among Family and Friends

Family and friends constituted a major support and trust network for almost all college students interviewed in this study in both Hawaii and Japan. Common characteristics and themes can be seen in the responses of students from Hawaii and Japan:

“[Family members] share with each other. We get our biggest support from each other. We like each other” (Female student interview, Reitaku University, January 15, 2009).

“I have a lot of friends and my husband is pretty good and my family is pretty supportive. Even though they don’t live here, I can just call them and cry for an hour or whatever” (Female student interview, University of Hawaii at Manoa, February 26, 2009).

The above-mentioned statements are representative of the sentiments voiced by the participants at Reitaku University and the University of Hawaii. According to the interview transcripts, social support and mutual trust play a universally important role in maintaining good health status and well-being.

Students at Reitaku University and the University of Hawaii particularly valued mutual trust and support provided in times of stress and depression. Even if students experienced family problems, stress, mental health problems, or problems with interpersonal relationships, they were more likely to rely upon the social capital and trust from new or existing relationships. For example:

“[My friends] are under a lot of stress, so it's pretty much ‘I’m there for you, you’re there for me’” (Female student interview, Reitaku University, January 20, 2009).

“Personally for us, there was a fairly stable relationship and environment and network of friends. ‘When I feel a lot of stress, my friends are there for me.’ ‘When they feel a lot of stress, I am there for them’” (Male student interview, University of Hawaii at Manoa, February 27, 2009).

#### 3.1.2 Support and Trust from Neighbors

Many college students at the University of Hawaii stated that their closest friends were also their neighbors and people from within their community. Thus, these students are more likely to have a good image of their neighbors and friends. Further, since they are more likely to trust people in their community, they would want to continue living in their community because of the strong ties stemming from social capital. For example:

“[I have] good friends, neighbors in my hometown. I live in [a] Japanese community in Honolulu… Like the time when I am making something to have at dinner, I provided some food to my neighbors. I feel [a] sense of community or belonging. I think trusting people in my community is very [common]” (Male student interview, University of Hawaii at Manoa, February 26, 2009).

Compared to students at the University of Hawaii, students at Reitaku University were less likely to trust people in their community or town. Consequently, Japanese students were less likely to be involved in community activities. The Japanese students’ distrust in their neighbors was evident in their responses:

“When I was child, I got along with [my] neighbors. However, not anymore… I do not receive any support and I do not trust my neighbors any more. I do not know who [the] neighbors [are that] live… close to my place. I know my parents sometimes communicate with [them], but not me” (Male student interview, Reitaku University, January 19, 2009).

Based on perceptions of social capital and trust, respondents from both samples were asked to report any support or help received from friends and family, any social groups they were a member of, and any communities in which they were involved. They were asked to think widely about the different types of support that they might have received over the previous year, including emotional, economic, and instrumental (help to know or do things).

The interview responses about the perception of trust in social capital revealed crucial important cultural differences between college students in Hawaii and Japan. In Japan, respondents reported few opportunities to build trust or to create social ties in order to obtain work or help when it was most needed. Factors such as the recent economic crisis and social problems contributed to Japanese college students experiencing uncertainty about their future, feelings of hopelessness, and a lack of successful role models. In contrast, while only 78 percent of students in Hawaii reported having negative feelings of daily life events, compared to 94 percent of Japanese students, 100 percent of students in both cultures reported positive feelings about their daily life events. This indicates that Japanese students are most likely to feel stress, distress, and suffer from depression due to a lack of social networks, support, trust, and motivation when it comes to investing human capital in their own health and well-being.

Although support and trust in a social capital network used to be pre-eminent initially, Japanese students reported that this was no longer true and these findings suggest that dark side perspectives about trust in social capital may be spreading in Japan. The role of social capital can have negative social consequences when it results in rigid in-groups. Otherwise, social capital can become weaker when individuals develop skepticism towards the help and supports they might receive from others and when they reduce their social involvement with others. When Japanese people faced challenges in the past, they were more likely to cooperate and resolve them because of the mutual trust in both social capital and each other. When the economic crisis and financial problems emerged in 2008, they caused a lot of retrenchment, and resulted in a higher level of mistrust in both social capital and each other. There is evidence that people with high levels of mistrust towards their community are less likely to gain access to employment opportunities or receive the help and support necessary to face challenges (Hibino et al., 2012; Japan Times, 2009; Tokuda, Fujii, Jimba, & Inoguchi, 2009).

This finding may also be supported by the fact that the recent rise in levels of mistrust in social capital are linked to higher levels of depression, which has resulted in an increased suicide rate in Japan, as noted by the *Japan Times* (2009). For example, more than 30,000 suicides have been committed each year in Japan since 1998; the 2009 data shows that the suicide rate from January through August was greater than in the corresponding months in 2008. The economic downturn and uncertainty about the future may have fuelled this trend. In 2008, the National Police Agency reported that depression was the most frequently cited factor for suicide, in 27.6 percent of cases. A government panel charged with investigating measures to prevent suicide stated that many suicide victims suffered from depression and were either dependent on alcohol or subsumed by a strong sense of guilt (Japan Times, 2009). In order to help prevent suicide, which is the main consequence of depression, further research is needed to clarify the mechanisms by which trust in social capital or mistrust in it affects depression and mental health and well-being from psychosocial and communication perspectives (Hibino et al., 2012; Japan Times, 2009; Tokuda et al., 2009).

Previous research has always focused on the sunny side of social capital. However, this study points out the mistrust in the “dark side of social capital” among Japanese college students. Thus, this study endeavors to pay attention to the “dark side of social capital” (Portes & Landolt, 1996). When examining the opposing possibilities leading to the positive side of social capital which is so often discussed in sociological and health-science research, it is important to look at a new factor, “tolerance,” the notion that the original social capital theory by Putnam (2002) failed to point out. In order to continue being democratic “joiners” in the public arena where heterogeneous worldviews, antagonistic partisans, and/or incompatible policy supporters are all present, people must be tolerant of different ideas and ideologies. Tolerance is not unrelated to trust, a well-discussed element of social capital, however, it is more directly relevant to people's attitudes toward social control (permissiveness) of others’ deeds than trust (Putnam, 2002).

When intolerance prevails, the social system runs the risk of transforming the sunny side of social capital into the dark side. Under such circumstances, social capital cannot work well and surveillance is needed at all times; antagonism prevails despite the existence of active voluntary associations. This “dark side of social capital” was first identified and indicated in early arguments Portes and Landolt (1996) and Putnam (2002), who also discussed this perspective directly, mainly in terms of tolerance, although in a quiet, optimistic tone (Hibino et al., 2012; Tokuda et al., 2009).

To summarize, Japanese society used to have an interchangeable abundance of social capital and resources, but these no longer appear to be available. This study, therefore, highlights the need to reinforce trust among people in order to reconstruct social capital. It is important to recognize available networks and support systems, bring about the rebuilding and reorganization of the school network system, social services, and roles to connect people and foster social capital. This study also suggests that, by rebuilding and reorganizing social network ties, job market access will increase and lead to less unemployment and a more effective use of human capital. If students can recognize and realize their strengths, values, social roles, and communication skills and invest these in human and social capital, they will be more likely to forge firm ties in the job market, strengthen the social capital base, and be rewarded with high human capital invested careers (Hibino et al., 2012; Tokuda et al., 2009).

High levels of social capital have traditionally been a salient factor among the Japanese because of their characteristics of egalitarianism, collectivism, or groupism, their Confucian heritage, ethnic homogeneity, and lifetime employment (Hibino et al., 2012; Tokuda et al., 2009). Moreover, provisions for public welfare encourage Japanese people to construct social capital for mutual aid (Lockhart, 2001). These socio-cultural characteristics may encourage mutual help and reliance among the Japanese rather than enhance their reliance on public welfare. They strengthen social integration and solidarity favorable to the in-group, but at the expense of the out-group. Distrust of the out-group and foreigners is a common characteristic of the Japanese people (Yamagishi, Kikuchi, & Kosugi, 1999). On the other hand, there will be a stable relationship among the in-group and social network (Yamagishi et al., 1999). Therefore, social capital, such as “old boys’ networks,” is demonstrated through its contribution to people's access to the Japanese labor market. Social capital plays a notable role in social control within Japan. Overall, social capital is consolidated in Japan; it is enduring, dependable, and functional, and contributes maximal benefits to success among Japanese people (Hibino et al., 2012; Tokuda et al., 2009).

The research showed that college students at the University of Hawaii were more likely than Japanese college students to invest in human capital to get a job. Although this could lead to a loss of social capital, the college students, mainly the local ones at the University of Hawaii, were more likely to think positively and demonstrate stronger behaviors such as self-esteem, self-confidence, self-enhancement, self-promotion, and independence than their Japanese counterparts. There could be a “sunny side of social capital” in Hawaii, but the interview data showed that the college students, mainly the local ones, were more likely to trust people within their community. Hawaii is a very small and unique island community compared to the mainland U.S. The island's historical background may be seen to influence its current social capital. Originally, people came to Hawaii to pursue jobs on the plantations. On arrival, although poor, they trusted and cooperated with each other to build and invest strongly and effectively in social human capital. These factors continue to influence the small island community and its sense of “sunny side of social capital;” the sense of belonging, sense of fairness, and sense of family (*“ohana”*) still prevails among the college students, specifically the ones from the local neighborhood at the University of Hawaii. Moreover, the college students mainly from the local community at the University of Hawaii may be seen to promote the “positive side of social capital” to increase networking among the number of people in their schools, community or society (Hibino et al., 2012; Tokuda et al., 2009).

The research interview posed questions about membership in communities, small groups, or societies in order to measure the current connections and gauge social capital. Specifically, many respondents from the University of Hawaii had religious affiliations. For example, “I’m Catholic” was a common response when asked about membership of religious groups, and “I live in the Hawaiian community” was a common response when asked about memberships in ethnic or cultural groups. Respondents considered memberships in parishes or specific religious prayer meeting groups to mean active participation in religious groups. On the other hand, college students at Reitaku University were less likely to be members of religious, ethnic, or cultural groups and they were also less likely to trust religious organizations due to cultural influences. Japanese students were also likely to recognize some religious beliefs and practices as appropriate traditional and cultural behaviors, rather than to identify themselves as followers of these beliefs or practices themselves (Hibino et al., 2012; Tokuda et al., 2009).

### 3.2 Lack of Support

Coping strategies, help, and support from interpersonal and institutional relationships, organizations, and institutions and the wider community are not always available or sufficient. Coping strategies, help, and support from interpersonal relationships, family members, and/or friends cannot always be relied upon, especially if they themselves are struggling under serious stress or problems which may be linked to severe depression and suicide (Japan Times, 2009). Some students at Reitaku University and at the University of Hawaii explained that the lack of support resulted in an escalation of their stress and health problems (Hibino et al., 2012; Tokuda et al., 2009). Although they did consider asking for help and support, they ended up facing their personal and social problems themselves. Even if some students wanted to talk to their friends, they were less likely to get the help and support they needed because they were less likely to rely on them, for example:

[For stress], “I just stored it up, I probably complained at home, maybe I was miserable at home. As far as using positive coping skills to deal with it and deal with it in a proactive way” (Female student interview, University of Hawaii at Manoa, on February 26, 2009).

“My parents came to here to work. Most of [the] people in my town are newcomers. Since we do not know most of [the] people in my town, we need to build up the connection to their community. Since there are many newcomers, I do not feel safe near the station or bus stop [near] my house” (Male student interview, Reitaku University, January 19, 2009).

### 3.3 Concept of Community and Trust

The individual interviews were prefaced with the statement “Now I would like to ask you some questions about your community,” and participants were presented with prompts to help them define their geographical communities. For students at both Reitaku University and the University of Hawaii, the term “community” was not defined for the respondents. Instead, defining their community was a task left to the respondents. However, the interviews revealed that many respondents did not have a clear concept of the meaning of the word “community.” It was a word hardly ever used spontaneously, and many respondents had difficulty defining what their community was, when asked. For example, nearly all of the respondents from rural areas equated “community” with “communal”—namely, the communal lands owned by the village for grazing cattle. In urban areas, however, half of the respondents interpreted “community” as people helping each other (Hibino et al., 2012; Tokuda et al., 2009).

The individual interviews were prefaced with the statement, “Can you trust the majority of people?” College students at the University of Hawaii were more likely to answer that they could easily trust people in their community. However, college students at Reitaku University were more likely to avoid the direct use of the concept of “trusting people in their community or trusting friends or family” because of cultural biases and differences. Even though they were more likely to agree to the concept of “trusting people, such as friends, family, or community members,” as culturally appropriate, they were less likely to directly express the concept of “trusting people.” Based on the interview scripts in Japan, “trust” was more likely to have many processes and mechanisms attached to it. Thus, the findings of this study may suggest that the word “trust” among the Japanese is more likely to have many layers, mechanisms, and interpretations from the viewpoints of cultural bias and differences (Hibino et al., 2012; Tokuda et al., 2009).

For example:

“Trust in Japanese is [a] very vague concept and meaning. As you know, I am not able to easily use the word ‘trust.’ Of course, I am trusting people. However, even though I am trusting people, avoiding [the] direct express [ion of] the word ‘trusting’ is very [common] in Japan” (Female student interview, Reitaku University, January 20, 2009).

## 4. Discussion and Conclusion

This qualitative interview investigation has provided a powerful tool for a more in-depth exploration of the associations and relationships among significant social capital and their impacts on the health and well-being of college students at the University of Hawaii at Manoa and Reitaku University. The findings of the in-depth interviews indicate the following. First, significant social capital—mainly perceptions of trust in social capital—have a big impact on health and well-being in both Hawaii and Japan. This study also found cultural differences in terms of trust in social capital and its effects on health and well-being. College students at the University of Hawaii at Manoa were more likely to trust people in their community and invest in social capital in order to obtain support and resources when they needed them. However, college students at Reitaku University were less likely to trust people in their community (and thus were more likely to mistrust people in their community) and have lower social capital investments (and hence, fewer opportunities to get the support and resources when needed). Losing trust and social capital were likely to produce social pressures, social anxiety, and social stress, ultimately resulting in higher levels of stress and lower levels of health and well-being. This is seen to finally culminate in a higher level of depression in terms of mental health and well-being while producing a higher rate of suicide as a result (Hibino et al., 2012; Tokuda et al., 2009).

This study showed how unclear and vague the concepts of “community” and “trust” were among college students. Students at the University of Hawaii at Manoa were more likely to clearly understand and capture the concept of “community.” Moreover, the comprehension of the term “trust” could be different because of cultural differences and the bias brought about by the Japanese language among college students at Reitaku University. Due to the cultural influence and biases, even though Japanese participants were likely to express their trust in people in their community, they were more likely to avoid directly expressing their “trust” of people in their community, as was the norm in terms of their traditional and culturally appropriate behavior. Moreover, this study also clarified the link between a lack of energy and fatigue in physical and psychological health. The interview data provides the psychological or communication mechanisms of severe depression and suicide in psychological and mental health. Future research is needed to clarify the mechanisms of depression and suicide from the view of psychological health, mental health and well-being (Hibino et al., 2012; Tokuda et al., 2009).

Overall, this study has reported the results of interviews designed to achieve a deeper understanding of the relationships between significant social capital such as social capital, and social environmental factors (e.g., socio-economic and socio-demographic statuses) and their impact on health and well-being and to achieve a deeper understanding of whether the relationships among social capital are stable in terms of health and well-being when examined across different cultures.

## 5. Claims and Implications

Qualitative in-depth interviews provided more thorough detail on significant social factors. The eleven main themes in this study are informed by views of: (1) perception of trust in social capital; (2) cultural differences in terms of trust in social capital and its effects relative to health and well-being; (3) unclear and vague concepts of “community” and “trust”; (4) linkage between lack of energy and fatigue in physical and psychological health; (5) differences in religious beliefs and practices; (6) an indistinct understanding of the concept of spirituality; (7) generational differences in religious beliefs; (8) perceptions of social security and safety; (9) personal lifestyles; (10) recycling and global warming issues; and (11) health insurance. Overall, social factors, such as social capital and other socio-environmental factors defined by the male and female college students at the University of Hawaii at Manoa and Reitaku University in Japan seem to center on eleven main themes.

## References

[ref1] Blum H. L (1981). Planning for health.

[ref2] Giordano G. N, Lindstrom M (2010). The impact of changes in different aspects of social capital and material conditions on self-rated health over time: a longitudinal cohort study. Social Science and Medicine.

[ref3] Hibino Y, Takaki J, Ogino K, Kambayashi Y, Hitomi Y, Shibata A, Nakamura H (2012). The relationship between social capital and self-rated health in a Japanese population: a multilevel analysis. Environment Health Preventive Medicine.

[ref4] Iwase T, Suzuki E, Fujiwara T, Takao S, Doi H, Kawachi I (2012). Do bonding and bridging social capital have differential effects on self-rated health? A community based study in Japan. Journal of Epidemiological Community Health.

[ref5] Japan Times (2009). Editorial: Depression and Suicide, Thursday, October 8 2009.

[ref6] Lau M, Li W (2011). The extent of family and school social capital promoting positive subjective well-being among primary school children in Shenzhen, China. Children and Youth Service Review.

[ref7] Lofors J, Sundquist K (2007). Low-linking social capital as a predictor of mental disorders: A cohort study of 4.5 million Swedes. Social Science and Medicine.

[ref8] Morgan A, Rivera F, Moreno C, Haglund B (2012). Does social capital travel? Influences on the life satisfaction of young people living in England and Spain. BMC Public Health.

[ref9] Oksanen T, Kouvonen A, Vahtera J, Virtanen M, Kivimäki M (2010). Prospective study of workplace social capital and depression: are vertical and horizontal components equally important?. Journal of Epidemiological Community Health.

[ref10] Poortinga W (2006). Social capital: an individual or collective resource for health?. Social Science and Medicine.

[ref11] Portes A, Landolt P (1996). Downside of social capital. The American Prospect.

[ref12] Putnam R. D (2002). Democracies in Flux: The Evolution of Social Capital in Contemporary Society.

[ref13] Summarch A. H. J (2011). Facilitating trust engenderment in secondary school nurse interactions with students. Journal of School Nursing.

[ref14] Suzuki E, Fujiwara T, Takao S, Subramanian S. V, Yamamoto E, Kawachi I (2010). Multi-level, cross-sectional study of workplace social capital and smoking among Japanese employees. BMC PublicHealth.

[ref15] Szreter S, Woolcock M (2004). Health by association? social capital, social theory, and the political economy of public health. International Journal of Epidemiology.

[ref16] Tokuda Y, Fujii S, Jimba M, Inoguchi T (2009). The relationship between trust in mass media and the healthcare system and individual health: evidence from the Asia Barometer Survey. BMC Med.

[ref17] Yamagishi T, Kikuchi M, Kosugi M (1999). Trust, gullibility, and social intelligence. Asian Journal of Social Psychology.

